# A portable automatic cough analyser in the ambulatory assessment of cough

**DOI:** 10.1186/1475-925X-9-17

**Published:** 2010-03-14

**Authors:** Malgorzata Krajnik, Iwona Damps-Konstanska, Lucyna Gorska, Ewa Jassem

**Affiliations:** 1 Palliative Care Department, Nicolas Copernicus University, Collegium Medicum in Bydgoszcz, Poland; 2 Department of Allergology, Chair of Lung Disease, Medical University of Gdansk, Poland

## Abstract

**Background:**

Cough is one of the main symptoms of advanced lung disease. However, the efficacy of currently available treatment remains unsatisfactory. Research into the new antitussives requires an objective assessment of cough.

**Methods:**

The aim of the study was to test the feasibility of a new automatic portable cough analyser and assess the correlation between subjective and objective evaluations of cough in 13 patients with chronic cough. The patients' individual histories, a cough symptom score and a numeric cough scale (1-10) were used as a subjective evaluation of cough and a computerized audio-timed recorder was used to measure the frequency of coughing.

**Results:**

The pre-clinical validation has shown that an automated cough analyser is an accurate and reliable tool for the ambulatory assessment of chronic cough. In the clinical part of the experiment for the daytime, subjective cough scoring correlated with the number of all cough incidents recorded by the cough analyser (r = 0.63; p = 0.022) and the number of cough incidents per hour (r = 0.60; p = 0.03). However, there was no relation between cough score and the time spent coughing per hour (r = 0.48; p = 0.1). As assessed for the night-time period, no correlation was found between subjective cough scoring and the number of incidents per hour (r = 0.29; p = 0.34) or time spent coughing (r = 0.26; p = 0.4).

**Conclusion:**

An automated cough analyser seems to be a feasible tool for the ambulatory monitoring of cough. There is a moderate correlation between subjective and objective assessments of cough during the daytime, whereas the discrepancy in the evaluation of night-time coughing might suggest that subjective analysis is unreliable.

## Background

Cough is one of the main symptoms of advanced lung disease. However, the efficacy of currently available treatment remains unsatisfactory. Growing knowledge of the pathophysiology of cough has resulted in the implementation of new therapeutic options and research on future potential treatment modalities [1-3]. Research into the efficacy of antitussives would, however, require the objective assessment of cough. Until now the evaluation of the frequency and severity of cough has been based mainly on patients' subjective reporting. According to the guidelines of the European Respiratory Society (ERS) and the American College of Chest Physicians (ACCP), treatment efficacy for chronic respiratory diseases should be assessed using an objective method of cough monitoring [4,5]. This very recent recommendation reflects observations from preliminary studies suggesting a poor and variable relationship between different subjective and objective measures of cough [6-11]. Despite great progress in the objective evaluation of cough, the introduction of equipment which would be useful in everyday clinical practice remains a challenge. Such portable, low-weight systems should be simple in their use and application, acceptable to patients and reliable.

**Figure 1:**
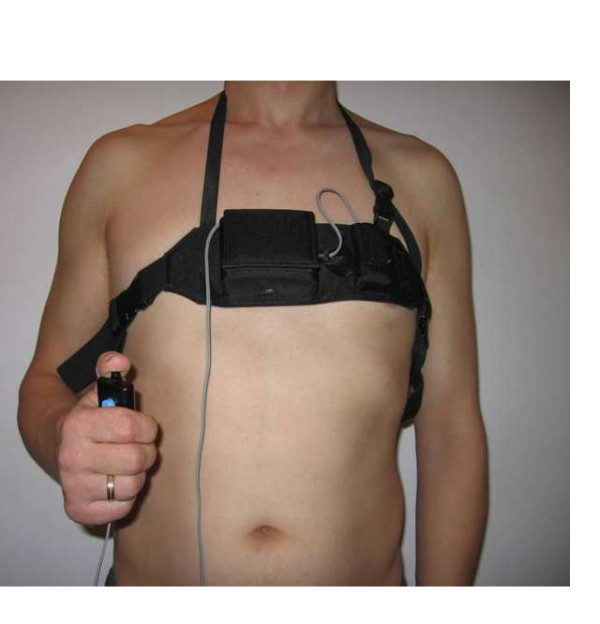
**Cough analyser**. The cough analyser registered all events continuously and automatically. Patients were handling a device which allowed them to mark certain important incidents, for example incidental dyspnoea.

In this study we first tested the feasibility of a new portable automatic cough analyser (CA) which could be applied to 24-hour monitoring in an ambulatory setting (Figure 1). We then used this method to determine the relation between subjective cough scores and objective monitoring by a CA. The aim of the clinical part of the study was to assess the relation between patients' evaluations and an objective measurement of cough frequency and time spent coughing.

## Methods

### Patients

Sixteen patients who had been treated at the Department of Pneumonology and Allergology at the Medical University of Gdansk, Poland, were included in the study (Table 1).

**Table 1:** Characteristics of the patients with respiratory diseases included in the study.

Nr	Gender F/M	Age (years)	Diagnosis	Dyspnoea (Borg scale)	Spirometry (% of predicted)			
					
					FEV1	FVC IN	FVC EX	FEV1/FVC
1	F	55	Asthma	3	106	104	108	83
2	F	43	Asthma	3	65	76	81	69
3	F	19	Asthma	-	105	109	110	83
4	M	34	Asthma	0	76	87	88	71
5	F	36	Asthma	4	101	102	109	79
6	F	58	Asthma	2	37	48	51	61
7	M	48	BHR	2	99	107	110	72
8	F	55	Asthma	5	125	137	143	74
9	F	56	Asthma	5	58	65	72	68
10	F	46	Asthma	-	104	104	110	81
11	F	56	Asthma	-	106	108	111	81
12	M	53	Asthma	1	96	121	122	62
13	F	48	BHR	2	107	122	127	72
14	F	31	BHR	1	118	122	126	81
15	M	51	Asthma	0	88	113	113	62
16	M	53	BHR	0.5	147	158	155	76

Patients 3, 10 and 11 did not complete the Borg scale; BHR - bronchial hyper-reactivity

The inclusion criteria were as follows: chronic cough defined according to the ERS [4], being over the age of 18, and giving written informed consent.

The exclusion criterion was asthma exacerbation. The study was approved by the Local Research Ethics Committee in Gdansk and patients' individual histories, a cough symptom score and a numeric cough scale (1-10) were used for a subjective evaluation of cough. Portable cough recording was commenced following lung function assessment by spirometry. In the case of ambulatory patients, these were allowed to go home and encouraged to wear the cough-recording device for 24 hours. Moreover, they were asked to self-report their cough during the day and night.

### Cough analyser

The cough analyser (CA) designed for the purpose of the study (MES, Kraków, Poland) was supplied with batteries (2 × AAA 2.4 V) and worn by patients in a special pouch around the thorax (Figure 1). A microphone with a reading frequency of 8-1200 Hz was attached to the pouch and localized in the lower part of the sternum, approximately 30 cm from the mouth. Signals from a microphone detector were continuously registered on a flash memory. After completing the analysis, data were transferred from the recorder to a personal computer and read automatically by the software (MES, Kraków, Poland), allowing for the analysis of particular parameters.

The CA was able to measure acoustic vibrations continuously and the signals being transferred to a recording of sound amplitude. The hardware for the CA allows for a 0.1-second resolution time in registering signals with a cut-off value of 20 dB, whereas the frequency range is 8-1200 Hz.

#### Pre-clinical part of the experiment: validation of the MES CA

The process of the MES CA validation consisted of three steps.

First - 6 healthy individuals voluntarily produced cough. A comparison of the agreement of manual counting by software using the MES Cough Analyser against counting by the hearing of cough incidents in real time was checked. The counting error (EC_h-m_) was assessed according to the following mathematical formula:

N_h _- number of incidents counted from hearing in real time

N_m _- number of incidents counted manually by the software

As N_h _= 1069 and N_m _= 1065, the EC_h-m _was equal to 0.37%.

Discrimination afforded by the manual counting of recordings with software for the MES CA was observed to be consistent with that achieved by counting in real time. Thus, the manual counting of the recordings was assessed as reliable and used in the next steps as the method of reference for automatic counting.

Second - the calibration of the cough analyser.

Six healthy volunteers coughed in their normal office environments at work (where external noise such as talking was present) (Table 2). The counting error (EC_a-m_) was evaluated for different parameters of automatic analysis, such as time (0.1 s or 1 s) and amplitude of discrimination (5%, 10%, 20% or 40%).

**Table 2:** The calibration of the cough analyser

Nr	N_h_	N_m_	Automatic counting by the cough analyser											
			
			T_d _= 0.1s A_d _= 10%		T_d _= 0.1s A_d _= 20%		T_d _= 0.1s A_d _= 40%		T_d _= 1 s A_d _= 20%		T_d _= 1 s A_d _= 10%		T_d _= 1 s A_d _= 5%	
			
			N_a_	EC_a-m _(%)	N_a_	EC_a-m _(%)	N_a_	EC_a-m_ (%)	N_a_	EC_a-m _(%)	N_a_	EC_a-m _(%)	N_a_	EC_a-m _(%)
1	164	162	178	9.88	175	8.03	151	6.79	156	3.7	166	**2.47**	182	12.35

2	174	177	194	9.61	190	7.35	164	7.35	169	4.52	182	**2.83**	199	12.43

3	171	173	191	10.41	185	6.94	161	6.94	165	4.62	178	**2.89**	194	12.14

4	199	197	217	10.15	212	7.61	182	7.61	188	4.57	204	**3.55**	223	13.20

5	134	132	145	9.85	142	7.58	121	8.33	126	4.55	135	**2.27**	147	11.36

6	227	224	247	10.27	240	7.14	207	7.59	214	4.46	232	**3.57**	255	13.84

Nr - number; N_h _- number of incidents counted from hearing in real time;

N_m _- number of incidents counted manually the software MES Cough Analyser;

N_a _- number of incidents automatically counted by cough analyser;

EC_a-m _- counting error; T_d _- discrimination time; A_d _- amplitude of discrimination.

EC_a-m _was assessed according to the following mathematical formula:

N_m _- number of incidents counted manually by the software MES Cough Analyser

N_a _- number of incidents automatically counted by the cough analyser

The results of the calibration tests showed that the optimal parameters of automatic analysis are time discrimination = 1 second and amplitude discrimination = 10%. This means that the recognition of a signal begins if the amplitude reaches 10% as well as disappearing at the level of 10%.

Using these parameters of automatic analysis means that EC_a-m _was equal to 2.93% (range 2.27-3.57), thus EC_a-m _was < 5%.

Third - automatic counting verified against that achieved via manual counting by the software MES Cough Analyser during prolonged monitoring (Table 3).

**Table 3:** The validation of the automatic measurement of cough against manual analysis by the MES Cough Analyser.

Nr	T [h]	N_a_	N_m_	TP	FN	FP	FP rate	TN	SENS [%]	SPEC [%]	AGREE [%]
1	8	1282	1238	1198	40	84	0.0035	23672	96.8	99.6	99.5

2	8	1343	1302	1277	25	66	0.0026	25285	98.1	99.7	99.6

3	8	1154	1120	1105	15	49	0.0019	25552	98.7	99.8	99.7

4	8	1705	1648	1620	28	85	0.0036	23692	98.3	99.6	99.5

5	8	1587	1543	1498	45	89	0.0035	25147	97.1	99.6	99.5

6	8	1782	1715	1657	58	125	0.0053	23680	96.6	99.5	99.3

7	16	2184	2114	2048	66	136	0.0027	50722	96.9	99.7	99.6

8	16	2730	2648	2573	75	157	0.0030	51725	97.2	99.7	99.6

9	16	3155	3041	2921	120	234	0.0045	51279	96.0	99.5	99.3

10	16	3286	3159	3024	135	262	0.0053	48928	95.7	99.5	99.2

11	20	3426	3308	3168	140	258	0.0041	62033	95.7	99.6	99.4

12	20	4311	4162	3976	186	335	0.0054	60805	**95.5**	99.5	99.2

13	20	4139	3984	3850	134	289	0.0046	61451	96.6	99.5	99.3

Nr - number of measurement period; T - duration of measurement in hours; N_m _- number of incidents counted manually by software for the MES Cough Analyser; N_a _- number of incidents automatically counted by cough analyser; TP - true positives = number of incidents detected by automatic counting and confirmed by manual analysis; FN - false negatives = number of incidents detected only by manual analysis (not by automatic counting); FP - false positives = number of incidents detected only by automatic analysis (not by manual counting); TN - true negatives = number of potential incidents not registered by automatic or manual counting (as the discrimination time was 1 second, a TN was counted as seconds of measurement without incidents registered either by automatic or manual analysis);

The thirteen measurements in healthy volunteers lasted a total of 172 hours.

The parameters of digital measurement accepted for signal detection were based on the results of the previous step of calibration: discrimination time = 1 second; minimal amplitude of incident = 10%. Thus, the software enabled registration of a 1-second incident as a cough (minimal cough duration = 1 second). A 1-second interval was required to discriminate between incidents. The software presented each incident as a percentage of the maximum amplitude achieved by an individual patient. The recognition of a signal began if the amplitude reached 10% and also disappeared at the level of 10%.

The results of the validation process enabled the calculation of sensitivity greater than 95% (median = 96.8; range 95.5-98.7), a specificity greater than 99% (median = 99.6; range 99.5-99.8), and a median false positive rate = 0.0036 (range 0.0019-0.0054) (Table 3).

Time was measured by the use of a quartz generator with a frequency of 3.62 MHz and an accuracy and stability of +/-50 ppm. Thus accuracy of the time measurement is very high, at the level of one-tenth of a millisecond. The filters of frequency used by us guaranteed the time of response < 0.01 second.

The hardware for the CA allows for a 0.1-second resolution time in registering signals. Thus, the hypothetical error related to the time measurement should be extremely low and acceptable.

#### Clinical experiment

To eliminate sounds of other than cough origin (i.e. extraneous noise) during the clinical part, we withdrew signals with an amplitude lower than 50% of the maximum amplitude obtained during cough. We quantified cough in terms of the number of incidents and the time spent coughing in seconds per hour; we analysed day and night periods separately.

### Subjective measures of cough

Patients were asked to self-assess their cough simultaneously with the CA monitoring. The evaluation included the intensity and duration of the symptom based on a Numerical Rating Scale (NRS: 1 = no cough to 10 = worst cough) and a cough scoring system recommended by the ERS [4,6].

### Statistical analysis

The validation of the CA revealed a sensitivity for discriminating between coughs equal to 95%. Based on clinical experience, we had assumed the sensitivity of the cough score to be 55%. We calculated that to obtain a power of 70% (p = 0.05) for the study we would need a sample size involving 16 patients [12].

The time for monitoring was arbitrarily divided into night (N) and day (D) (from 22.00 to 6.00 and the time remaining, respectively). The steps for the calculation included: (1) counting the number of cough incidents during D and N separately; and (2) calculating the mean number of incidents per hour for D and N. These mean values were subsequently used for further analysis of correlations between particular features. The same was calculated for time spent coughing. Statistical analysis was conducted using a licensed version of statistical software: STATISTICA PL 5.0 for Windows. The distribution of variables using the Shapiro-Wilk test was abnormal, therefore non-parametric statistical tests were chosen. A Wilcoxon rank test was performed to assess the differences between particular parameters. Spearman correlations were used to examine the relationship between subjective and objective measures of cough.

## Results

Sixteen patients with chronic cough were included in the CA assessment (11 females; median age 49.5 years [range 19-58]) (Table 1). Among these patients, three did not finish the subjective assessment. Thus a statistical assessment of the correlation between the objective and subjective cough evaluations was conducted for 13 patients (Table 4).

**Table 4:** Objective and subjective parameters of cough

Nr	Study duration (h)	Time spent coughing per hour			Number of incidents per hour			Longest interval between incidents (min)	Cough scoring		NRS 1-10
					
		Total (sec)	Day (sec)	Night (sec)	Total	Day	Night		Day	Night	
1	24	17	12	27	63	31	128	85	5	4	7

2	24	47	67	7	51	70	13	88	4	3	4

3	17.5	20	30	9	56	72	38	277	-	-	-

4	24	40	16	89	61	21	142	112	3	0	4

5	24	12	15	7	37	44	25	168	4	2	6

6	22.5	1	1	0	2	3	0	622	1	0	3

7	20	18	28	4	40	59	11	95	3	1	4

8	20.5	5	9	0	13	22	1	284	4	3	5

9	24	34	51	2	71	104	4	153	5	3	9

10	21.5	49	75	4	105	160	12	117	-	-	-

11	15	52	78	29	113	194	41	60	-	-	-

12	22	8	12	2	16	22	5	129	0	2	1

13	19	15	24	2	29	47	4	101	3	2	7

14	23.5	4	7	0	3	4	0	601	3	2	5

15	17	0	0	0	0	0	0	-	0	0	1

16	20	16	26	1	33	53	2	95	3	0	8

Cough scoring recommended by the ERS [6]. Cough scoring during daytime: 0 = no cough during the day; 1 = cough for one short period; 2 = cough for more than two short periods; 3 = frequent coughing, which did not interfere with usual daytime activities; 4 = frequent coughing, which did interfere with usual daytime activities; 5 = distressing coughs most of the day. Cough scoring during night-time: 0 = no cough during the night; 1 = cough on waking only; 2 = waking once or early due to cough; 3 = frequent waking due to cough activities; 4 = frequent coughs most of the night; 5 = distressing coughs preventing any sleep.

### Compatibility

The CA was easy to use and highly acceptable to the patients, apart from two (patients 12 and 13) who removed it early due to discomfort during the night. Eight other patients decided for themselves to shorten the time of monitoring due to occupational obligations.

### Analysis of objective cough monitoring

Among the 16 patients included in the analysis, the median duration time of objective cough monitoring (the entire study period) was 21 hours and 45 minutes (range 15-24 hours) (Table 4). The median of cough incidents for the whole study period was 38.5 per hour, with a wide range of 0-113. The number of incidents was higher during the day than the night-time period. The median number of incidents per hour during the day was 45.5 (range 0-194); at night this was 8 (range 0-142; p = 0.04, Wilcoxon rank test). Despite the lack of a significant relation between the number of cough events per hour during D and N (r = 0.47; p = 0.063), there was a moderate correlation between the number of all cough incidents during D and N (r = 0.53; p = 0.034): patients who coughed more during the day also coughed more at night (Table 5).

**Table 5:** Correlation between different parameters of cough

	Spearman correlation						
	
	Number of coughs per hour			Time spent coughing per hour (sec/h)			Cough score for a night period
		
	Total	Day	Night	Total	Day	Night	
Number of incidents per hour during daytime			0.47 (p = 0.064)				

Time spent coughing per hour (sec/h) during daytime						**0.60 (p = 0.014)**	

Cough intensity by the NRS	0.52 (p = 0.066)			0.37 (p = 0.21)			

Cough score for a day period		**0.60 (p = 0.029)**			0.48 (p = 0.1)		**0.77 (p = 0.002)**

Cough score for a night period			0.29 (p = 0.34)			0.26 (p = 0.4)	

The median time spent coughing for the study group was 16.5 seconds per hour with a wide range between patients (0-52). Time spent coughing was higher during the day than at night. The median time spent coughing during the day was 20 seconds per hour (range 0-78), whereas at night this was 3 seconds per hour (range 0-89; p = 0.02, Wilcoxon rank test). There was a moderate correlation between time spent coughing per hour during D and N (r = 0.60; p = 0.015).

### Analysis of subjective measurement

Thirteen patients completed the subjective evaluation form (Table 4). The median cough score during the day was 3 (range 0-5) and was higher than at night (median 2, range 0-4; p = 0.02; Wilcoxon rank test). There was a moderate correlation between cough scoring for D and N (r = 0.77; p = 0.002). Patients with a high cough score for the day tended to assess night coughing similarly (Table 5).

### Correlation between subjective and objective measures

**Figure 2:**
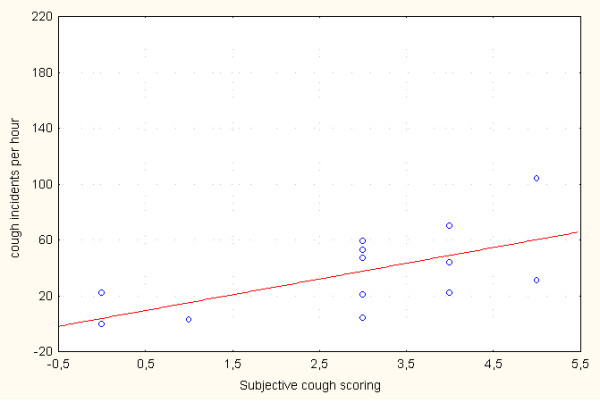
**Correlation between subjective cough scoring and the number of cough incidents per hour assessed for one daytime period (r = 0.60; p = 0.029)**.

**Figure 3:**
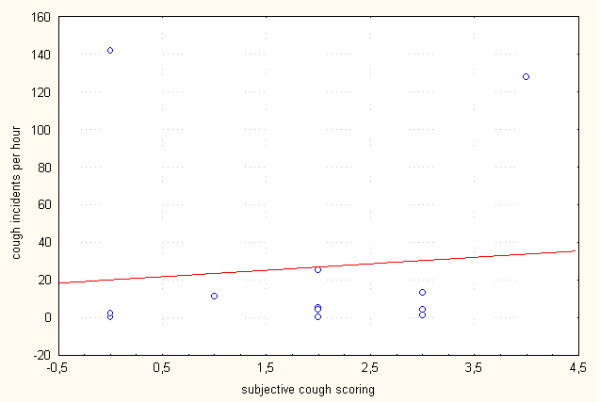
**Correlation between subjective cough scoring and the number of cough incidents per hour assessed for one night-time period (r = 0.29; p = 0.34)**.

For the daytime, subjective cough scoring correlated moderately with the number of all cough incidents (r = 0.63; p = 0.022) and the number of cough incidents per hour (r = 0.60; p = 0.029) (Table 5 and Figure 2). However, for the daytime period there was no significant correlation between cough score and the time spent coughing per hour (r = 0.48; p = 0.1). As assessed for the night-time period, there was no significant correlation between subjective cough scoring and all studied objective parameters, such as the number of incidents per hour (r = 0.29; p = 0.34) (Figure 3) or time spent coughing per hour (r = 0.26; p = 0.4). There was no significant correlation between the NRS and either cough incidents per hour (r = 0.52; p = 0.066) or time spent coughing per hour (r = 0.37; p = 0.21).

## Discussion

In the pre-clinical assessment our new portable automatic cough analyser seemed to be a simple and practical but accurate and reliable method for the objective prolonged monitoring of cough in an ambulatory setting. Our pilot clinical study compared subjective and objective evaluations of cough in patients with chronic cough. The results showed a moderate correlation between cough scoring and the number of cough incidents measured with the CA during the day, whereas there was a lack of correlation between these parameters at night.

Until now the relationships between different subjective and objective measures of cough have not been clarified and have been controversial in different trials [9-11]. In children with recurrent cough, 24-hour cough counts correlated moderately with cough scores assessed through the use of a colour-coded chart and a Visual Analogue Scale [7]. When the observation period was divided into day- and night-time, subjective night-time reporting of cough by children was unreliable [13-17]. Therefore, some authors have suggested that night-time cough should be removed from children's symptom diaries [16,18]. In adult patients with chronic obstructive pulmonary disease, subjective measures of cough correlate moderately with time spent coughing both during the day and at night [19]. By contrast, in patients with asthma or chronic dry cough subjective reporting correlates with cough counts only in patients with chronic dry cough and only during the day [6]. In the present study, we demonstrate a moderate correlation between subjective cough scoring and the number of cough incidents per hour measured with a CA for the daytime. However, no such correlation was found for the night-time period. Thus, despite the main limitation of our study due to the small sample size, our results support the observation that nocturnal cough might be unreliably reported in adults. An explanation for this phenomenon is difficult. Cough has been shown to be significantly influenced by the psychological status of the subjects [20,21]. We might hypothesize that the negative impact on quality of life from night cough is significant and leads to an overestimation of its severity. The other possible explanation is that patients transfer their daytime evaluation to the night-time assessment. Moreover, they might poorly and subjectively rate cough as a symptom both by day and night.

Recently, the need for integrated recording systems for cough assessment has been stressed, particularly in the clinical evaluation of drug efficacy [4,5]. There is still a need to differentiate between cough and non-cough sound [13,22-25]. In our study, the localization of the low-volume unidirectional contact microphone helped to eliminate extraneous noise and increase the chance that only high-amplitude sounds occurring close to the microphone would be detected. Our pilot study is an attempt to find the optimal means of reliable chronic cough assessment by using a practical and simple method.

It is worth mentioning that a group from Leicester, UK, introduced an automated system for the 24-hour monitoring of cough frequency. Their studies have proved to be significant, with repeatable differences seen in cough frequency between patients with chronic cough and healthy controls [26]; they have further validated their system against manual counts obtained by a trained observer [27]. Thus, they confirm that the automated counting of cough events is a reliable and objective method.

However, it is likely that automated cough monitoring is both device and method specific; therefore, the findings of previous studies cannot be extrapolated to new devices.

The unsatisfactory effectiveness of currently available antitussives, particularly in patients with advanced lung disease, has provoked increased interest in the development of some novel approaches. One of these is to reach opioid receptors in the trachea-bronchial tract using nebulised opioids [28] or other targets involved in cough, including transient receptor potential vanilloid-1 antagonists, selective cannabinoid agonists, maxi-K channel openers and purinergic receptor antagonists [for a review of this see [29]].

Clinical trials on new antitussive drugs would require the thorough objective assessment of the intensity and frequency of cough, which means that introducing portable devices such as a CA might be of clinical relevance.

## Conclusions

We conclude that appropriate night-time cough evaluation requires both subjective and objective monitoring. The severity of cough defined by a patient may not represent its frequency and duration when measured objectively. The use of ambulatory cough analysers should not be limited to research but might be helpful in clinical practice, particularly when symptomatic treatment is the main or only purpose of the approach.

## Competing interests

The authors declare that they have no competing interests.

## Authors' contributions

MK and IDK were involved in the design of the study protocol, in the patients enrolment and examination, in the analysis and interpretation of data and writing the paper. IDK performed a statistical analysis. LG took a part in the patients enrolment to the study, in a data collection and analysis, and revised the manuscript critically. EJ made substantial contribution to conception and design of the study protocol, analysis and interpretation of data, coordination of the trial and helped to draft the manuscript. All authors read and approved the final manuscript.

## References

[B1] Andre E Gatti R Trevisani M Preti D Baraldi PG Patacchini R Geppetti P Transient receptor potential ankyrin receptor 1 is a novel target for pro-tussive agents Br J Pharmacol 2009 158 1621 1628 10.1111/j.1476-5381.2009.00438.x 19845671 PMC2795228

[B2] Kamei J Yoshikawa Y Saitoh A Effect of N-arachidonoyl-(2-methyl-4-hydroxyphenyl) amine (VDM11), an anandamine transporter inhibitor, on capsaicin-induced cough in mice Cough 2006 2 2 10.1186/1745-9974-2-2 16623933 PMC1448189

[B3] Dicpinigaitis PV Potential future therapies for the management of cough: ACCP evidence-based clinical practice guidelines Chest 2006 129 284 286 10.1378/chest.129.1_suppl.284S 16428720

[B4] Morice AH Fontana GA Belvisi MG Birring SS Chung KF Dicpinigaitis PV Kastelik JA McGarvey LP Smith JA Tatar M Widdicombe J ERS Task Force. ERS guidelines on the assessment of cough Eur Respir J 2007 29 1256 1276 10.1183/09031936.00101006 17540788

[B5] Irwin RS Assessing cough severity and efficacy of therapy in clinical research Chest 2006 129 232S 237S 10.1378/chest.129.1_suppl.232S 16428716

[B6] Hsu JY Stone RA Logan-Sinclair RB Worsdell M Busst CM Chung KF Coughing frequency in patients with persistent cough: assessment using a 24 hour ambulatory recorder Eur Respir J 1994 7 1246 1253 10.1183/09031936.94.07071246 7925902

[B7] Chang AB Phelan PD Robertson CF Roberts RG Sawyer SM Relation between measurements of cough severity Arch Dis Child 2003 88 57 60 10.1136/adc.88.1.57 12495964 PMC1719282

[B8] Li AM Tsang TWT Chan DFY Lam HS So HK Sung RY Fok TF Cough frequency in children with mild asthma correlated with sputum neutrophil count Thorax 2006 61 747 750 10.1136/thx.2005.050815 16670174 PMC2117083

[B9] Decalmer S Webster D Kelsall A McGuinness K Woodcock A Smith J Chronic cough: how do cough reflex sensitivity and subjective assessments correlate with objective cough counts during ambulatory monitoring? Thorax 2007 62 329 334 10.1136/thx.2006.067413 17101736 PMC2092471

[B10] Kelsall A Decalmer S Webster D Brown N McGuinness K Woodcock A Smith J How to quantify coughing: correlations with quality of life in chronic cough Eur Respir J 2008 32 175 179 10.1183/09031936.00101307 18287128

[B11] Birring SS Fleming T Matos S Raj AA Evans DH Pavord ID The Leicester Cough Monitor: preliminary validation of an automated cough detection system in chronic cough Eur Respir J 2008 31 1013 1018 10.1183/09031936.00057407 18184683

[B12] Altman DG Gore SM, Altman DG How large a sample? Statistics in practice 1982 London, UK; British Medical Association

[B13] Matos S Birring SS Pavord ID Evans DH Detection of cough signals in continuous audio recordings using hidden Markov models IEEE Trans Biomed Eng 2006 53 1078 1083 10.1109/TBME.2006.873548 16761835

[B14] Falconer A Oldman C Helms P Poor agreement between reported and recorded nocturnal cough in asthma Pediatr Pulmonol 1993 15 209 211 10.1002/ppul.1950150405 8469572

[B15] Archer LNJ Simpson H Night cough counts and diary card scores in asthma Arch Dis Child 1985 60 473 474 10.1136/adc.60.5.473 4015154 PMC1777323

[B16] Chang AB Newman RG Carlin JB Phelan PD Robertson CF Subjective scoring of cough in children: parent-completed vs child-completed diary cards vs an objective method Eur Respir J 1998 11 462 466 10.1183/09031936.98.11020462 9551755

[B17] Hamutcu R Francis J Karakoc F Bush A Objective monitoring of cough in children with cystic fibrosis Pediatr Pulmonol 2002 34 331 335 10.1002/ppul.10174 12357476

[B18] McKenzie S Silverman M Clinical features and their assessment Childhood asthma and other wheezing disorders 1995 London: Chapman & Hall Medical 175 200

[B19] Smith J Owen E Earis J Woodcock A Cough in COPD: correlation of objective monitoring with cough challenge and subjective assessments Chest 2006 130 379 385 10.1378/chest.130.2.379 16899835

[B20] Dales RE Spitzer WO Schechter MT Suissa S The influence of psychological status on respiratory symptom reporting Am Rev Respir Dis 1989 139 1459 1463 2729753 10.1164/ajrccm/139.6.1459

[B21] Rietveld S Van Beest I Everaerd W Psychological confounds in medical research: the example of excessive cough in asthma Behav Res Ther 2000 38 791 800 10.1016/S0005-7967(99)00099-6 10937427

[B22] Coyle MA Keenan DB Henderson LS Watkins ML Haumann BK Mayleben DW Wilson MG Evaluation of an ambulatory system for the quantification of cough frequency in patients with chronic obstructive pulmonary disease Cough 2005 1 3 10.1186/1745-9974-1-3 16270923 PMC1277005

[B23] Murata A Ohota N Shibuya A Ono H Kudoh S New non-invasive automatic cough counting program based on 6 types of classified cough sounds Intern Med 2006 45 391 397 10.2169/internalmedicine.45.1449 16617191

[B24] Barry SJ Dane AD Morice AH Walmsley AD The automatic recognition and counting of cough Cough 2006 2 8 10.1186/1745-9974-2-8 17007636 PMC1601963

[B25] Smith J Ambulatory methods for recording cough Pulm Pharmacol Ther 2007 20 313 318 10.1016/j.pupt.2006.10.016 17161969

[B26] Birring SS Matos S Patel RB Prudon B Evans DH Pavord ID Cough frequency, cough sensitivity and health status in patients with chronic cough Respir Med 2006 100 1105 1109 10.1016/j.rmed.2005.09.023 16266801

[B27] Matos S Birring SS Pavord ID Evans DH An automated system for 24-h monitoring of cough frequency: the leicester cough monitor IEEE Trans Biomed Eng 2007 54 1472 1479 10.1109/TBME.2007.900811 17694868

[B28] Krajnik M Podolec Z Siekierka M Sykutera M Pufal E Sobanski P Makarewicz R Neef C Punt N Zylicz Z Morphine inhalation in cancer patients. A comparison of different nebulization techniques using pharmacokinetic, spirometric and gasometric parameters J Pain Symptom Manage 2009 19783397 10.1016/j.jpainsymman.2009.03.008

[B29] Barnes PJ The problem of cough and development of new antitussives Pulm Pharmacol Ther 2007 20 416 422 10.1016/j.pupt.2006.11.001 17189707

